# Evidence of Japanese Encephalitis Virus Exposure in Feral Pig Populations in North Queensland, Australia

**DOI:** 10.1155/tbed/7087198

**Published:** 2026-09-28

**Authors:** Andrew M. Adamu, Cadhla Firth, Bruce Gummow, Andrew J. Hoskins, Roslyn I. Hickson, Antonio Di Rubbo, Kalpana Agnihotri, David T. Williams, Paul F. Horwood

**Affiliations:** ^1^ Discipline of Veterinary Science, College of Science and Engineering, James Cook University, Townsville and Cairns, Australia, jcu.edu.au; ^2^ Australian Institute of Tropical Health and Medicine, James Cook University, Townsville and Cairns, Australia, jcu.edu.au; ^3^ Center for Tropical Biosecurity, James Cook University, Townsville and Cairns, Australia, jcu.edu.au; ^4^ Commonwealth Scientific and Industrial Research Organisation, Townsville, Australia, csiro.au; ^5^ Department of Production Animals, Faculty of Veterinary Science, University of Pretoria, Pretoria, South Africa, up.ac.za; ^6^ Northern Australia Indigenous Land and Sea Management Alliance, Darwin, Northern Territory, Australia; ^7^ School of the Environment, University of Queensland, Brisbane, Queensland, Australia, uq.edu.au; ^8^ CSIRO, Australian Centre for Disease Preparedness, Geelong, Victoria, Australia, csiro.au

**Keywords:** Australia, feral pigs, Japanese encephalitis virus, Queensland

## Abstract

Several animal species, including horses and pigs, can be infected with Japanese encephalitis virus (JEV). Feral pigs (*Sus scrofa*) are considered an effective amplifying host for JEV in natural habitats. In this study, 265 dried blood spots were collected from feral pigs in four selected North Queensland local government areas, and antibodies against JEV were detected using a competitive enzyme‐linked immunosorbent assay (cELISA). The results showed that 26.4% (70/265) of the feral pigs had antibodies specific to JEV. We found a significant difference in the seroprevalence of JEV in the Wet Tropics (*p* < 0.05), compared to the Dry Tropics region of North Queensland. The results indicate that feral pigs in North Queensland have been exposed to JEV, suggesting that they may play an important role as an amplifying host. These findings may inform the planning of mitigation measures through a multisectoral One Health approach.

## 1. Background

Japanese encephalitis virus (JEV) is an important neuroinvasive, zoonotic, mosquito‐borne RNA virus that causes Japanese encephalitis (JE) [1]. It belongs to the species *Orthoflavivirus japonicum*, family Flaviviridae, and is the type member of the JE serological complex along with the closely related Murray Valley encephalitis virus (MVEV), St Louis encephalitis virus (SLEV), and West Nile virus (WNV) [2]. Five genotypes of JEV (G‐I, II, III, IV, and G‐V) have been described [3]. The enzootic transmission cycle of JEV involves pigs (domestic and feral), which act as major amplifying hosts for JEV, alongside ardeid birds, which serve as maintenance or reservoir hosts [4, 5]. In temperate zones, JE epidemics occur mostly in the summer period, while in tropical zones, JEV is endemic, and transmission occurs year‐round at a lower incidence [6]. A recent meta‐analysis of JEV in pigs in Mainland China estimated an overall prevalence of 35.2% [7]. The authors observed that the prevalence was highest during 2010–2015, at 53.4%, possibly reflecting increased rainfall and favorable temperatures that promoted mosquito proliferation, with the lowest prevalence of 2.5% observed in temperate climates. The distribution of this disease is predominantly in Asia and Western Pacific nations, culminating in nearly 100,000 cases of viral encephalitis each year, with a substantial burden among children under the age of 14 years [8]. The clinical presentation of JE is marked by considerable morbidity and mortality, with ~50% of those who survive likely to develop neurological sequelae [9].

Regular occurrences of JEV have been recorded in the Torres Strait Islands and Northern Peninsula Area (NPA) of Cape York in North Queensland [10], where the virus was first isolated in Australia [11]. Since then, viral activity previously thought to be restricted to North Queensland [11] has subsequently spread to the southern part of the country [6, 12]. Previously in Australia, JEV outbreaks caused by genotype II (G‐II) were reported in 1995 and 1998 and genotype I (G‐I) outbreaks in 2000 and 2004 [10, 11, 13]. The origin of the 1995 and 1998 outbreaks was considered to be Papua New Guinea; genetically identical strains of JEV were isolated from *Culex sitiens* subgroup mosquitoes trapped in the Western Province of PNG [14], while the origin of the 2000 and 2004 incursions remains unknown. Serological surveillance of livestock in the Torres Strait Islands and NPA has also provided evidence for seasonal activity of JEV between 2005 and 2021 in these locations [15]. The 2021–2022 outbreak of JEV in southern Australia was associated with genotype IV, a lineage not previously detected in the country. The outbreak was substantial, with JEV infection detected in pigs on more than 80 farms across the southeastern states and more than 40 confirmed human cases, including seven deaths [5]. Public health surveillance indicates that JEV transmission has persisted in southern Australia since the initial outbreak, with sporadic human infections and associated fatalities. During the outbreak, *Culex annulirostris*, *C. gelidus*, and *C. quinquefasciatus* were implicated as potential mosquito vectors [16, 17].

Transmission dynamics are influenced by a complex interplay among the virus, vectors, and hosts, driven by multifactorial environmental, genetic, and ecological variables [18, 19]. Factors that can influence JEV circulation include land use changes, translocation of infected hosts, and bird migration [20]. In India, pig farming in urban areas was associated with JEV circulation in peri‐urban and urban areas; likewise, rice farming resulted in increased standing water, thereby increasing vector presence [21, 22]. Together, these drivers highlight the need to understand JEV dynamics within specific regional contexts, including northern Australia. JEV emergence is influenced by diverse mosquito vectors and alternative hosts. While *Culex tritaeniorhynchus* is the principal vector in Southeast Asia, secondary vectors including *Culex annulirostris*, *Culex gelidus*, and *Culex quinquefasciatus* may contribute to transmission in Australia [23–25]. Alternative hosts, including chickens and potentially sheep, may sustain viral amplification, whereas dead‐end hosts, such as cattle, dogs, and horses, may reduce transmission through dilution effects, highlighting complex ecological drivers of JEV maintenance and spread [20, 26].

The Wet Tropics region in Northern Australia, located in northern Queensland, has a very large population of feral pigs and the environmental and ecological conditions are suitable for high levels of JEV circulation [5, 21]. The region is characterized by a rugged, high‐rainfall landscape dominated by tropical rainforest, alongside mangroves, wet sclerophyll forests, and floodplain wetlands [27, 28]. Perennial streams, upland refugia, complex vegetation structure, and seasonal hydrology support exceptional biodiversity, high local endemism, and strong altitudinal turnover in species, while also increasing vulnerability to climate change [29]. Despite the importance of the Wet Tropics as a suitable habitat for feral pigs, their role as a potential reservoir for this arboviral pathogen remains unclear. Here, we investigated the circulation of JEV antibodies in feral pigs across the Dry and Wet Tropics of North Queensland.

## 2. Materials and Methods

### 2.1. Study Areas/Design

A total of 265 dried blood spots were randomly collected from apparently healthy feral pigs in North Queensland between 2023 and 2025. The samples were collected through collaborations with hunters, farmers, long‐standing local council pest management initiatives, and a hunting festival. Four local government areas were chosen for the study, two from the Dry Tropics (Charters Towers Regional Council and Townsville City Council) and two from the Wet Tropics (Hinchinbrook Shire Council and Cassowary Coast Regional Council [CCRC]) (Figure 1). Feral pigs were aged by estimation using physical characteristics (body size and weight) and dental eruption and categorized as piglets (1–14 kg), juveniles (16–24 kg), and adults (>24 kg) [30]. Blood was applied to Whatman qualitative filter paper (Grade 1) and air‐dried to remove moisture. Age, sex, location, and date of collection were recorded for all samples. The specimens were placed in zippered storage bags with desiccant to maintain low humidity and transported to the Australian Institute of Tropical Health and Medicine (AITHM) in Townsville, Queensland, and stored at −20°C to preserve sample integrity until analysis.

**Figure 1:**
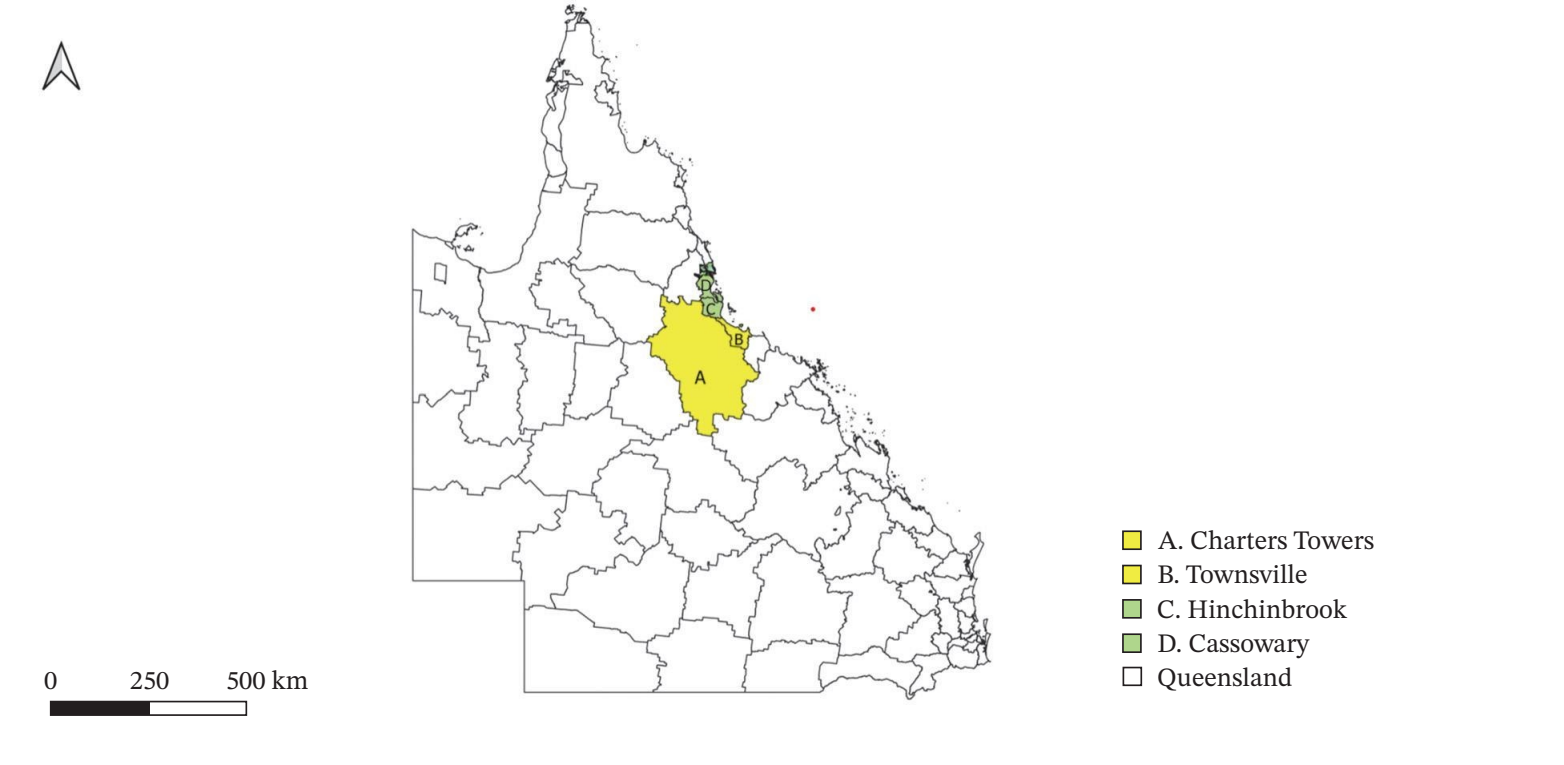
Map of Queensland highlighting the Dry (yellow color) and Wet (green color) Tropics of North Queensland.

### 2.2. Sample Size Calculation

The sample size was calculated using the formula proposed by Thrusfield [31]: *n = Z* ^2^ *pq*/*d* ^2^. Here, *n* represents the minimum sample size, *Z* denotes the z‐score corresponding to a 95% confidence interval (CI) (i.e., 1.96), *p* signifies the anticipated prevalence, *q* is defined as (1 − *p*), and *d* is the desired 5% precision. Given limited published data on JEV in Australian feral pigs, we used the seroprevalence estimate of 15.6% reported by Yap et al. [32] in Singapore. This yielded a minimum sample size of 202. To account for uncertainty, an additional buffer of 30% was incorporated, given that no JEV antibodies (0%) were detected by Hanna et al. [11] in Cape York, Queensland. Ultimately, we determined the sample size to be 263 and subsequently rounded it to 265.

### 2.3. Serum Extraction From Dried Blood Spot Filter Papers

Before analysis, filter papers (DBS) were allowed to equilibrate for 30 min and processed as described by Adamu et al. [33]. Six hundred microlitres of phosphate‐buffered saline (PBS), pH 7.4 (1X) (Life Technologies Corporation, USA) was added to each 1.8 mL cryogenic microtube containing filter paper to elute the serum. The eluted sera were labeled and transported on ice packs to the Australian Centre for Disease Preparedness, Geelong, Victoria, for serological analysis.

### 2.4. Detection of Serum Antibodies to JEV Using Competitive Enzyme‐Linked Immunosorbent Assay (cELISA)

JEV antibody was detected using a cELISA, which measures total JEV antibody levels in porcine [34, 35], bovine [36], and equine [37] serum by assessing the relative degree of inhibition of monoclonal antibody 989 (mAb 989). Sera were tested at a 1:10 dilution in the ELISA diluent (PBST + 1% skim milk powder) using cELISA to detect total antibodies to JEV, as described previously [34, 35]. MicrCoplates were read at an optical density set at 450 nm. ELISA results were interpreted as follows: percentage inhibition (PI) ≥ 50% = positive; 40%–49.9% = inconclusive; and <40% = negative. Only positive results were considered for subsequent analyses.

### 2.5. Statistical Analysis

All data were analyzed in R version 4.3.3 (R Development Core Team, Vienna, Austria), and statistical significance was set at *p*  < 0.05. The proportion of seropositivity of JEV in feral pigs was calculated, and the variability was assessed using the Clopper‐Pearson 95% CI. Distribution of feral pig characteristics and seropositivity was presented as frequencies and percentages. Associations between feral pig seropositivity and predictor variables (age, sex, year, LGAs, and weather conditions) were assessed using univariate logistic regression. PI data were further analyzed in Python (v2025.3.1.1) along with the pandas and seaborn libraries. Positive samples with ≥50% but less than 80% inhibition were further considered positive, while those with PI ≥80% were deemed highly positive, with the mean inhibition threshold set at ≥80%. For visualization purposes, the data were presented as box‐and‐whisker plots across age, sex, and location (Figure 2). Individual data points were superimposed and distinguished by color coding (≥80% in red; <80% in black), while a horizontal line at the 80% mark served as the mean threshold for positive PI values.

**Figure 2:**
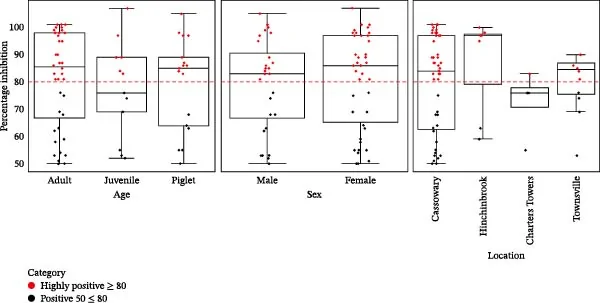
Box‐and‐whisker plot distribution of JEV‐positive samples with percentage of inhibition across age, sex and location.

## 3. Results

An overall JEV seroprevalence of 26.4% (70/265) was detected in this study. Seropositivity according to feral pig developmental stages was observed as follows: adult 26.9% (40/149), juvenile 19.4% (13/67), and piglets 34.7% (17/49). Piglets were ~1.5 times more likely to be seropositive to JEV than adults, though this difference was not statistically significant (OR = 1.45, 95% CI: 0.72–2.87; *p* = 0.29). Seropositivity based on sex was 24.8% (28/113) in boars and 27.6% (42/152) in sows, with no significant difference between sexes (OR = 1.16, 95% CI: 0.67–2.03; *p* = 0.60). Temporal distribution of JEV across sampling years showed that feral pigs sampled in 2023 (34.3%; 23/67) had a higher seropositivity rate than those sampled in 2024 (25.5%; 47/184), and none were positive in 2025 (0/14). However, the 2025 sample was small (*n* = 14), and collection sites differed between years, limiting direct comparison.

Results from the sampled administrative local government areas indicate that 43.1% (47/109) of feral pigs sampled in the CCRC area were seropositive for JEV antibodies. Seroprevalence was significantly lower in Hinchinbrook (OR = 0.2, 95% CI: 0.08–0.46) and Townsville (OR = 0.21, 95% CI: 0.1–0.43) compared to CCRC. In both seasons and administrative regions, estimates were accompanied by wide CIs, likely reflecting small sample sizes or extreme prevalences in some groups. Results from the Wet Tropics indicate that one‐third of sampled feral pigs were seropositive and were nearly three times more likely to be exposed to JEV than those in the Dry Tropics (Table 1).

**Table 1:** Univariate logistic regression of feral pig characteristics and predictors associated with JEV seropositivity in North Queensland.

Variable	*n*	Positive	Prev. % (95% CI)	*p*‐Value	OR (95% CI)
Overall	265	70	26.42 (21.21–32.16)	—	—
Age					
Adult	149	40	26.85 (19.92–34.71)	Ref	—
Juvenile	67	13	19.4 (10.76–30.89)	0.24	0.67 (0.31–1.3)
Piglet	49	17	34.69 (21.67–49.64)	0.29	1.45 (0.72–2.87)
Sex					
Male	113	28	24.78 (17.14–33.78)	Ref	—
Female	152	42	27.63 (20.7–35.46)	0.60	1.16 (0.67–2.03)
Year					
2023	67	23	34.33 (23.15–46.94)	0.17	1.52 (0.83–2.77)
2024	184	47	25.54 (19.41–32.48)	Ref	—
2025	14	0	0 (0–23.16)	—	—
Location					
Cassowary	109	47	43.12 (33.67–52.95)	Ref	—
Charters Towers	17	4	23.53 (6.81–49.9)	0.14	0.41 (0.11–1.23)
Hinchinbrook	53	7	13.21 (5.48–25.34)	**0.0004**	0.2 (0.08–0.46)
Townsville	86	12	13.95 (7.42–23.11)	**0.00003**	0.21 (0.1–0.43)
Tropics					
Dry	103	16	15.53 (9.15–24)	Ref	—
Wet	162	54	33.33 (26.13–41.16)	**0.00017**	2.72 (1.48–5.21)

*Note:* Ref = reference category used for comparison analysis. Bold values indicate statistical significance.

Of the 70 positive samples, 41/70 (58.6%) had antibody responses above the 80% inhibition threshold. The lower quartile (Q1) of the PI was 64%, the median quartile (Q2) was 84%, and the upper quartile (Q3) was 97%. No outliers beyond the whiskers were identified; individual PI values ranged from 50% to 107% [34] (Supporting Information 1). Furthermore, the percentage of inhibition exhibited variability across factors such as age, sex, and geographic region.

## 4. Discussion

This study provides the first evidence of widespread JEV exposure among feral pigs across the Wet Tropic region of Queensland, Australia. Following the 2021–2022 outbreak that marked JEV’s expansion onto the Australian mainland, the widespread distribution and high abundance of feral pigs raised concerns regarding their potential role in maintaining viral transmission and complicating surveillance and control efforts [38]. Despite this potential epidemiological importance, surveillance data for JEV in feral pigs in Australia remain limited as most investigations have focused on domestic pigs during outbreaks or routine surveillance activities [39].

In the present study, an overall seroprevalence of 26.4% was detected across four local government areas in northern Queensland, indicating substantial exposure of feral pig populations to JEV in the region. This level of seropositivity is consistent with the ecological and geographic context of northern Australia. The proximity of northern Queensland to the Indo‐Papuan conduit represents an ongoing biosecurity challenge as this region is recognized as a major pathway for the introduction of exotic pathogens, including JEV, into the Australian mainland [10, 19, 40]. Additionally, the tropical climate of northern Queensland, characterized by high temperatures and humidity, supports persistent mosquito populations and enhances the transmission dynamics of mosquito‐borne pathogens.

Comparisons with other JEV‐endemic regions further contextualize these findings. Previous studies have reported JEV seroprevalence in feral pigs ranging from 3.7% to 64.6% in Japan [41, 42], 66% in Korea [43], and 15.16% in Singapore [32]. The seroprevalence observed in this study falls within the range reported across Asia but exceeds the estimates from Singapore and some regions of Japan [32, 41]. Conversely, higher seroprevalence values reported in Korea and parts of Japan [42, 43] are more than twice those observed in this study, illustrating the potential for JEV to reach high levels of endemicity in feral pig populations under favorable ecological conditions. Together, these findings suggest that feral pigs in northern Queensland could serve as an important component of the JEV transmission cycle and highlight the need for continued wildlife surveillance to better understand the epidemiology and potential persistence of JEV in Australia.

Consistent with previous studies identifying feral pigs as important amplifying hosts of JEV [43, 44], our findings revealed significantly higher seroprevalence among feral pigs in the Wet Tropics compared to the Dry Tropics of northern Queensland. The Wet Tropics contains more than 16 distinct rainforest ecosystems, including the Daintree Rainforest, which provides a favorable habitat for feral pigs and supports diverse wildlife communities [45]. In this region, approximately one‐third of sampled feral pigs had detectable antibodies to JEV, indicating substantial viral exposure. Several ecological factors may explain the higher seroprevalence observed in the Wet Tropics. The region supports extensive wetlands, high annual rainfall, and persistently warm temperatures—conditions that favor the proliferation of mosquito vectors, particularly *Culex* species, and increase opportunities for host–vector contact [46]. In contrast, the Dry Tropics are characterized by more seasonal rainfall and fewer permanent wetland habitats, which may limit vector abundance and reduce transmission intensity. Together, these ecological differences likely contributed to the observed spatial heterogeneity in JEV exposure.

Additionally, feral piglets exhibit higher apparent seroprevalence (34.69%) and were ~1.5 times more likely to be seropositive for JEV than feral adult pigs in our study. The higher apparent seroprevalence observed in piglets may reflect persistence of maternally derived antibodies, recent exposure following waning maternal immunity, or a combination of these processes. Maternal antibody wanes between 1 and 3 months after weaning [47] but may persist for months [48, 49]. The detection of JEV in feral pigs underscores their epidemiological importance as free‐ranging hosts that may contribute to virus maintenance and amplification outside domestic piggeries. Together, these ecological and host‐related factors likely explain the elevated exposure risk observed in piglets, highlighting the contribution of feral pig populations to JEV persistence and spillover risk in North Queensland.

The Wet Tropics also lie along the East Asian Australasian Flyway (EAAF), a major migratory pathway for waterbirds extending from the Taimyr Peninsula in Russia and Alaska to Australia and New Zealand. Along this flyway are several countries such as China, Japan, Korea, and nations in Southeast Asia and the Western Pacific nations where JEV is endemic and which collectively support habitats for an estimated 50 million migratory waterbirds [50]. These birds depend on highly productive wetlands as stopover sites during migration. The wetlands of the Wet Tropics may therefore function as important staging habitats, making it plausible that migratory waterbirds contribute to the introduction or maintenance of JEV in the region. Waterbirds are known to develop high viraemia following infection and may act as reservoir hosts capable of sustaining enzootic transmission in the presence of competent mosquito vectors [7, 19].

Environmental conditions in northern Australia are broadly comparable to those of endemic regions in Southeast Asia, supporting the potential for year‐round JEV activity. Nevertheless, all reported outbreaks in Australia between 1995 and 2022 occurred between December and May [51]. This pattern broadly aligns with the seasonal period of arbovirus detection in Australia, typically between October and April, which coincides with peak mosquito activity [52]. We could not perform seasonal analysis from our study due to the sampling strategy employed, which may have resulted in an uneven distribution of samples across seasons (with more samples collected in autumn) and may have introduced bias into the seasonal estimates. Climate change may further influence these transmission dynamics. The increasing frequency and intensity of extreme climatic events, including flooding, cyclones, and prolonged La Niña conditions, are likely to expand suitable habitats for mosquito vectors and reservoir hosts. Such changes may enhance the persistence of transmission cycles and increase the risk of the establishment of sustained endemic transmission of JEV [53].

This study identified areas of northern Queensland that were not previously prioritized for JE vaccination [53]. Animal surveillance is a critical component of early detection of JEV activity, often identifying viral circulation before the occurrence of human cases and enabling timely public health responses. In Australia, JEV outbreaks have occurred across extensive geographic regions with relatively sparse human populations, where serological surveillance of piggeries and mosquito monitoring have been central to determining the extent of viral transmission [7]. Pigs act as amplifying hosts for JEV, producing high levels of viraemia that facilitate onward transmission to other hosts in the presence of competent mosquito vectors [54]. These findings highlight the need to further investigate the burden of human infection within the study regions of northern Queensland. An improved understanding of human exposure would support evidence‐based procurement and targeted allocation of JE vaccines and guide public health strategies and risk communication for populations at increased risk [17].

Several limitations should be considered when interpreting these findings. Unequal sample sizes across categories resulted in wide CIs for some estimates. In addition, the cross‐sectional design limited the ability to infer causal relationships between ecological factors and JEV exposure. The 2025 finding of zero positives is difficult to interpret given the small sample size and differences in collection sites between years and should not be taken as evidence of reduced JEV circulation. Continued sampling with broader geographic coverage will be needed to assess temporal trends in feral pig seroprevalence. Serological cross‐reactivity with other flaviviruses may also occur, as previously observed [11, 34]. High levels of serological cross‐reactivity occur between orthoflaviviruses, especially among members of the JEV serocomplex, and interpretation of serological test results should therefore be undertaken in this context [34]. Northern Australia is known to be hyperendemic for other JEV serocomplex members, such as MVEV and WNV‐Kunjin, and the results found in this study may reflect prior exposure of feral pigs to these viruses. Additional parallel testing for antibodies to these viruses to assess cross‐reactivity was beyond the scope and resources of this study. Furthermore, the study population consisted of feral pigs, which were not vaccinated and readily seroconvert following infection [55], supporting the interpretation that the detected antibodies reflect natural exposure to JEV or a related orthoflavivirus. Despite these limitations, the results provide consistent evidence for the circulation of JEV antibodies in the feral pig populations in northern Queensland.

The substantial spatial and temporal heterogeneity in JEV exposure observed here, with geographic location and climate zone emerging as key determinants, underscores the value of integrated One Health frameworks that incorporate humans, wildlife, livestock, mosquito vectors, and environmental monitoring [17]. Strengthening such frameworks will improve the early detection of viral activity and support targeted interventions in ecologically suitable regions. The detection of widespread JEV exposure in feral pigs across North Queensland highlights the ecological complexity of viral transmission in Australia and the potential role of wildlife in sustaining transmission cycles. Coordinating wildlife and vector monitoring with human health surveillance will be essential for improving early warning capacity, guiding vaccination policy, and mitigating the risk of sustained or expanding JEV transmission under changing climatic conditions.

## Author Contributions

Conceptualization: Andrew M. Adamu, Cadhla Firth, Andrew J. Hoskins, Roslyn I. Hickson, Bruce Gummow, and Paul F. Horwood. Methodology: Andrew M. Adamu, Antonio Di Rubbo, Kalpana Agnihotri, David T. Williams, and Paul F. Horwood. Sample collection: Andrew M. Adamu and Paul F. Horwood. Resources: Roslyn I. Hickson, Kalpana Agnihotri, David T. Williams, and Paul F. Horwood. Writing – original draft preparation: Andrew M. Adamu. Writing – review and editing: Andrew M. Adamu, Roslyn I. Hickson, Bruce Gummow, Kalpana Agnihotri, David T. Williams, and Paul F. Horwood. Visualization: Bruce Gummow, Roslyn I. Hickson, David T. Williams, and Paul F. Horwood. Supervision: Cadhla Firth, Andrew J. Hoskins, Roslyn I. Hickson, Bruce Gummow, and Paul F. Horwood.

## Funding

The authors received no funding for this work.

## Disclosure

All authors have read and agreed to the published version of the manuscript.

## Ethics Statement

This work received clearance from the Queensland Department of Primary Industries (Biosecurity Queensland) and a waiver from the James Cook University Animal Ethics Committee Number 22611.

## Conflicts of Interest

The authors declare no conflicts of interest.

## Supporting Information

Additional supporting information can be found online in the Supporting Information section.

## Supporting information


**Supporting Information** Percentage inhibition ranking from lowest to highest of JEV seropositivity in feral pigs in North Queensland.

## Data Availability

Data included in this study will be made available upon reasonable request.

## References

[1] Lord J. S. , Gurley E. S. , and Pulliam J. R. , Rethinking Japanese Encephalitis Virus Transmission: A Framework for Implicating Host and Vector Species, PLoS Neglected Tropical Diseases. (2015) 9, no. 12, 10.1371/journal.pntd.0004074, e0004074. PMC4686064 26657648

[2] Mackenzie J. S. , Barrett A. D. T. , and Deubel V. , The Japanese Encephalitis Serological Group of Flaviviruses: A Brief Introduction to the Group, Japanese Encephalitis and West Nile Viruses, 2002, Springer, 1–10. 10.1007/978-3-642-59403-8_1 12082984

[3] Solomon T. , Ni H. , Beasley D. W. C. , Ekkelenkamp M. , Cardosa M. J. , and Barrett A. D. T. , Origin and Evolution of Japanese Encephalitis Virus in Southeast Asia, Journal of Virology. (2003) 77, no. 5, 3091–3098, 10.1128/JVI.77.5.3091-3098.2003. PMC149749 12584335

[4] Oliveira A. R. S. , Cohnstaedt L. W. , Noronha L. E. , Mitzel D. , McVey D. S. , and Cernicchiaro N. , Perspectives Regarding the Risk of Introduction of the Japanese Encephalitis Virus (JEV) in the United States, Frontiers in Veterinary Science. (2020) 7, 10.3389/fvets.2020.00048, 48. PMC7019853 32118069

[5] Furlong M. , Adamu A. M. , and Hoskins A. , et al.Japanese Encephalitis Enzootic and Epidemic Risks Across Australia, Viruses. (2023) 15, no. 2, 10.3390/v15020450, 450. PMC9962251 36851664

[6] Mackenzie J. S. , Williams D. T. , van den Hurk A. F. , Smith D. W. , and Currie B. J. , Japanese Encephalitis Virus: The Emergence of Genotype IV in Australia and Its Potential Endemicity, Viruses. (2022) 14, no. 11, 10.3390/v14112480, 2480. PMC9698845 36366578

[7] Liu X. T. , Jiang L. D. , and Lin Y. T. , et al.Prevalence of Japanese Encephalitis in Pigs in Mainland China During 2000–2024: A Systemic Review and Meta-Analysis, Frontiers in Veterinary Science. (2025) 12, 10.3389/fvets.2025.1534114, 1534114. PMC11842376 39989665

[8] Quan T. M. , Thao T. T. N. , Duy N. M. , Nhat T. M. , and Clapham H. , Estimates of the Global Burden of Japanese Encephalitis and the Impact of Vaccination From 2000-2015, eLife. (2020) 9, 10.7554/eLife.51027, e51027. PMC7282807 32450946

[9] Turtle L. , Easton A. , and Defres S. , et al.More Than Devastating—Patient Experiences and Neurological Sequelae of Japanese Encephalitis, Journal of Travel Medicine. (2019) 26, no. 7, 10.1093/jtm/taz064, taz064. PMC6792067 31504712

[10] Hanna J. N. , Ritchie S. A. , and Phillips D. A. , et al.An Outbreak of Japanese Encephalitis in the Torres Strait, Medical Journal of Australia. (1996) 165, 256–260. 10.5694/j.1326-5377.1996.tb124960.x 8816682

[11] Hanna J. N. , Ritchie S. A. , and Phillips D. A. , et al.Japanese Encephalitis in North Queensland, Australia, 1998, Medical Journal of Australia. (1999) 170, no. 11, 533–536, 10.5694/j.1326-5377.1999.tb127878.x. 10397044

[12] Gentle M. , Wilson C. , and Cuskelly J. , Feral Pig Management in Australia: Implications for Disease Control, Australian Veterinary Journal. (2022) 100, no. 10, 492–495, 10.1111/avj.13198. PMC9796028 35851468

[13] Ritchie S. A. , Phillips D. , Broom A. , Mackenzie J. , Poidinger M. , and Hurk A. V. D. , Isolation of Japanese Encephalitis Virus From *Culex annulirostris* in Australia, The American Journal of Tropical Medicine and Hygiene. (1997) 56, no. 1, 80–84. 10.4269/ajtmh.1997.56.80 9063367

[14] Johansen C. A. , Van den Hurk A. F. , and Ritchie S. A. , et al.Isolation of Japanese Encephalitis Virus From Mosquitoes (Diptera: Culicidae) Collected in the Western Province of Papua New Guinea, 1997-1998, American Journal of Tropical Medicine and Hygiene. (2000) 62, no. 5, 631–638, 10.4269/ajtmh.2000.62.631. 11289676

[15] Sikazwe C. , Neave M. J. , and Michie A. , et al.Molecular Detection and Characterisation of the First Japanese Encephalitis Virus Belonging to Genotype IV Acquired in Australia, PLoS Neglected Tropical Diseases. (2022) 16, no. 11, 10.1371/journal.pntd.0010754, e0010754. PMC9721490 36409739

[16] Furlong M. , Adamu A. , and Hickson R. I. , et al.Estimating the Distribution of Japanese Encephalitis Vectors in Australia Using Ecological Niche Modelling, Tropical Medicine and Infectious Disease. (2022) 7, no. 12, 10.3390/tropicalmed7120393, 393. PMC9782987 36548648

[17] Skinner E. B. , Sartorius B. , and Furuya-Kanamori L. , et al.Ecological Suitability of Japanese Encephalitis Virus in Australia: A Modelling Analysis of Vector-Host Transmission Dynamics to Potential Spillover in Humans, PLOS Neglected Tropical Diseases. (2025) 19, no. 11, 10.1371/journal.pntd.0013722, e0013722. PMC12654935 41248205

[18] Oliveira A. R. S. , Cohnstaedt L. W. , and Cernicchiaro N. , Japanese Encephalitis Virus: Placing Disease Vectors in the Epidemiologic Triad, Annals of the Entomological Society of America. (2018) 111, no. 6, 295–303, 10.1093/aesa/say025.

[19] Mulvey P. , Duong V. , and Boyer S. , et al.The Ecology and Evolution of Japanese Encephalitis Virus, Pathogens. (2021) 10, no. 12, 10.3390/pathogens10121534, 1534. PMC8704921 34959489

[20] Levesque Z. A. , Walsh M. G. , Webb C. E. , Zadoks R. N. , Brookes V. J. , and Bowen R. A. , A Scoping Review of Evidence of Naturally Occurring Japanese Encephalitis Infection in Vertebrate Animals Other Than Humans, Ardeid Birds and Pigs, PLoS Neglected Tropical Diseases. (2024) 18, no. 10, 10.1371/journal.pntd.0012510, e0012510. PMC11482687 39365832

[21] Walsh M. G. , Pattanaik A. , and Vyas N. , et al.High-Risk Landscapes of Japanese Encephalitis Virus Outbreaks in India Converge on Wetlands, Rain-Fed Agriculture, Wild Ardeidae, and Domestic Pigs and Chickens, International Journal of Epidemiology. (2022) 51, no. 5, 1408–1418, 10.1093/ije/dyac050. PMC9557850 35355081

[22] Raju K. H. K. , Sabesan S. , and Rajavel A. R. , et al.A Preliminary Study to Forecast Japanese Encephalitis Vector Abundance in Paddy Growing Area, With the Aid of Radar Satellite Images, Vector-Borne and Zoonotic Diseases. (2016) 16, no. 2, 117–123, 10.1089/vbz.2014.1757. 26824289

[23] Pearce J. C. , Learoyd T. P. , Langendorf B. J. , and Logan J. G. , Japanese Encephalitis: The Vectors, Ecology and Potential for Expansion, Journal of Travel Medicine. (2018) 25, no. Suppl_1, S16–S26, 10.1093/jtm/tay009. 29718435

[24] van den Hurk A. F. , Skinner E. , Ritchie S. A. , and Mackenzie J. S. , The Emergence of Japanese Encephalitis Virus in Australia in 2022: Existing Knowledge of Mosquito Vectors, Viruses. (2022) 14, no. 6, 10.3390/v14061208, 1208. PMC9231386 35746679

[25] Kurucz N. , Roberts A. P. , and Weir R. P. , et al.Molecular Detection of Japanese Encephalitis Virus and Murray Valley Encephalitis Virus in Mosquitoes: Implications for Disease Transmission in the Northern Territory, Australia, Journal of Vector Ecology. (2025) 50, no. 2, 82–92, 10.52707/1081-1710-50.2-82. 41420810

[26] Zhang H. , Li D. , and Zheng J. , et al.Sheep Serve as Amplifying Hosts of Japanese Encephalitis Virus, Increasing the Risk of Human Infection, Science Advances. (2025) 11, no. 20, 10.1126/sciadv.ads7441, eads7441. PMC12083518 40378218

[27] Metcalfe D. J. and Ford A. J. , Floristics and Plant Biodiversity of the Rainforests of the Wet Tropics, Living in a Dynamic Tropical Forest Landscape, 2008, Wiley, 123–132.

[28] Bradford M. G. , Metcalfe D. J. , Ford A. , Liddell M. J. , and McKeown A. , Floristics, Stand Structure and Aboveground Biomass of a 25-ha Rainforest Plot in the Wet Tropics of Australia, Journal of Tropical Forest Science. (2014) 26, no. 4, 543–553.

[29] Connolly N. M. , Christidis F. , McKie B. , Boyero L. , and Pearson R. , 12 Diversity of Invertebrates in Wet Tropics Streams: Patterns and Processes, Living in a Dynamic Tropical Forest Landscape, 2009, Blackwell Publishing, 161–177.

[30] Twigg L. E. , Lowe T. , Martin G. , and Everett M. , Feral Pigs in North-Western Australia: Basic Biology, Bait Consumption, and the Efficacy of 1080 Baits, Wildlife Research. (2005) 32, no. 4, 281–296, 10.1071/WR04110.

[31] Thrusfield M. V. , Veterinary Epidemiology, 2007, 3rd edition, Blackwell Science Ltd., 228–242.

[32] Yap G. , Lim X. F. , and Chan S. , et al.Serological Evidence of Continued Japanese Encephalitis Virus Transmission in Singapore Nearly Three Decades After End of Pig Farming, Parasites & Vectors. (2019) 12, no. 1, 10.1186/s13071-019-3501-0, 244. PMC6525359 31101069

[33] Adamu A. M. , Hoskins A. J. , Firth C. , Gummow B. , Hickson R. I. , and Horwood P. F. , Serological Evidence of Akabane, Bluetongue, and Bovine Ephemeral Fever Virus Exposure in Feral Water Buffaloes From Northern Australia, Viruses. (2026) 18, no. 3, 10.3390/v18030363, 363. PMC13030519 41902271

[34] Williams D. T. , Daniels P. W. , Lunt R. A. , Wang L. F. , Newberry K. M. , and Mackenzie J. S. , Experimental Infections of Pigs With Japanese Encephalitis Virus and Closely Related Australian Flaviviruses, American Journal of Tropical Medicine and Hygiene. (2001) 65, no. 4, 379–387, 10.4269/ajtmh.2001.65.379. 11693888

[35] Hevoho E. , Di Rubbo A. , Yombo A. K. , Williams D. T. , Gummow B. , and Brookes V. J. , Seroprevalence of Japanese Encephalitis in Pigs in Central Province, Papua New Guinea, Vector-Borne and Zoonotic Diseases. (2026) . 10.1177/15303667261442507 41973116

[36] Pant G. R. , Lunt R. A. , Rootes C. L. , and Daniels P. W. , Serological Evidence for Japanese Encephalitis and West Nile Viruses in Domestic Animals of Nepal, Comparative Immunology, Microbiology and Infectious Diseases. (2006) 29, no. 2-3, 166–175, 10.1016/j.cimid.2006.03.003. 16697904

[37] Lam K. H. K. , Ellis T. M. , and Williams D. T. , et al.Japanese Encephalitis in a Racing Thoroughbred Gelding in Hong Kong, Veterinary Record. (2005) 157, no. 6, 168–173, 10.1136/vr.157.6.168. 16085723

[38] Williams C. R. , Webb C. E. , Higgs S. , and van den Hurk A. F. , Japanese Encephalitis Virus Emergence in Australia: Public Health Importance and Implications for Future Surveillance, Vector-Borne and Zoonotic Diseases. (2022) 22, no. 11, 529–534, 10.1089/vbz.2022.0037. 36354964

[39] Moore K. T. , Mangan M. J. , and Linnegar B. , et al.Australian Vertebrate Hosts of Japanese Encephalitis Virus: A Review of the Evidence, Transactions of the Royal Society of Tropical Medicine and Hygiene. (2025) 119, no. 3, 189–202, 10.1093/trstmh/trae079. PMC11887621 39451055

[40] Horwood P. F. , McBryde E. S. , Peniyamina D. , and Ritchie S. A. , The Indo-Papuan Conduit: A Biosecurity Challenge for Northern Australia, Australian and New Zealand Journal of Public Health. (2018) 42, no. 5, 434–436, 10.1111/1753-6405.12808. 30088687

[41] Nidaira M. , Taira K. , and Itokazu K. , et al.Survey of the Antibody Against Japanese Encephalitis Virus in Ryukyu Wild Boars (*Sus scrofa* Riukiuanus) in Okinawa, Japanese journal of infectious diseases. (2007) 60, no. 5, 309–311. 17881875

[42] Nidaira M. , Kyan H. , and Taira K. , et al.Survey of Japanese Encephalitis Virus in Pigs and Wild Boars on Ishigaki and Iriomote Islands in Okinawa, Japan, Epidemiology and Infection. (2014) 142, no. 4, 856–860, 10.1017/S0950268813001611. PMC9151112 23830350

[43] Yang D.-K. , Kim H.-H. , and Hyun B.-H. , et al.Detection of Neutralizing Antibody Against Japanese Encephalitis Virus in Wild Boars of Korea, Journal of Bacteriology and Virology. (2012) 42, no. 4, 353–356, 10.4167/jbv.2012.42.4.353.

[44] Park S. L. , Huang Y. J. S. , and Lyons A. C. , et al.Infection of Feral Phenotype Swine With Japanese Encephalitis Virus, Vector-Borne and Zoonotic Diseases. (2023) 23, no. 12, 645–652, 10.1089/vbz.2023.0030. PMC10698774 37672628

[45] Koichi K. , Cottrell A. , and Sangha K. K. , Implications of Invasive Alien Species for Rainforest Tourism: A Case Study on Feral Pigs in Australia’s Wet Tropics World Heritage Area, Rainforest Tourism, Conservation and Management, 2014, Routledge, 259–274.

[46] Dale P. E. R. and Knight J. M. , Wetlands and Mosquitoes: A Review, Wetlands Ecology and Management. (2008) 16, no. 4, 255–276, 10.1007/s11273-008-9098-2.

[47] Scherer W. F. , Moyer J. T. , Izumi T. , Gresser I. , and McCown J. , Ecologic Studies of Japanese Encephalitis Virus in Japan, The American Journal of Tropical Medicine and Hygiene. (1959) 8, no. 6, 698–706. 10.4269/ajtmh.1959.8.698 14442656

[48] Burke D. S. , Tingpalapong M. , Ward G. S. , Andre R. , and Leake C. J. , Intense Transmission of Japanese Encephalitis Virus to Pigs in a Region Free of Epidemic Encephalitis, Southeast Asian Journal of Tropical Medicine and Public Health. (1985) 16, no. 2, 199–206. 2999995

[49] Hale J. H. , Lim K. A. , and Colless D. H. , Investigation of Domestic Pigs as a Potential Reservoir of Japanese B Encephalitis Virus on Singapore Island, Annals of Tropical Medicine & Parasitology. (2016) 51, no. 4, 374–379, 10.1080/00034983.1957.11685827. 13498656

[50] East Asian–Australasian Flyway Partnership , About the Flyway, East Asian–Australasian Flyway Partnership, n.d., Accessed 20 Apr. 2026 https://www.eaaflyway.net.

[51] Di Francesco J. , Choeung R. , and Peng B. , et al.Comparison of the Dynamics of Japanese Encephalitis Virus Circulation in Sentinel Pigs Between a Rural and a Peri-Urban Setting in Cambodia, PLoS Neglected Tropical Diseases. (2018) 12, no. 8, 10.1371/journal.pntd.0006644, e0006644. PMC6107123 30138381

[52] Veliz A. R. , Kane S. , and Glynn-Robinson A. , Emergence of Locally Acquired Japanese Encephalitis Virus in Australia: A National Case Series, Communicable Diseases Intelligence. (2025) . 10.33321/cdi.2025.49.005 39837615

[53] Knope K. , Doggett S. L. , and Jansen C. C. , et al.Arboviral Diseases and Malaria in Australia, 2014–15: Annual Report of the National Arbovirus and Malaria Advisory Committee, Communicable Diseases Intelligence. (2019) 43, 1–71, 10.33321/cdi. 30982295

[54] McGuinness S. L. , Lau C. L. , and Leder K. , The Evolving Japanese Encephalitis Situation in Australia and Implications for Travel Medicine, Journal of Travel Medicine. (2023) 30, no. 2, 10.1093/jtm/taad029, taad029. PMC10075061 36869722

[55] Kikuchi F. , Hayashi A. , and Yamada K. , et al.The Role of Wild Boar as Host of Japanese Encephalitis Virus in the Absence of Domestic Pigs, Viruses. (2024) 16, no. 8, 10.3390/v16081273, 1273. PMC11360753 39205248

